# Blood viscosity associated with stroke mechanism and early neurological deterioration in middle cerebral artery atherosclerosis

**DOI:** 10.1038/s41598-023-36633-z

**Published:** 2023-06-09

**Authors:** Ho Geol Woo, Hyug-Gi Kim, Kyung Mi Lee, Sang Hee Ha, HangJin Jo, Sung Hyuk Heo, Dae-il Chang, Bum Joon Kim

**Affiliations:** 1 Department of Neurology, Kyung Hee University College of Medicine, Kyung Hee University Hospital, Seoul, Korea; 2 Department of Radiology, Kyung Hee University College of Medicine, Kyung Hee University Hospital, Seoul, Korea; 3 Department of Neurology, Gil Medical Center, Gachon University, Incheon, Korea; 4 Department of Mechanical Engineering and Division of Advanced Nuclear Engineering, POSTECH, Pohang, Gyeongbuk Korea; 5 Department of Neurology, Asan Medical Center, University of Ulsan, College of Medicine, Song-Pa, PO Box 145, Seoul, 138-600 Korea

**Keywords:** Cerebrovascular disorders, Stroke

## Abstract

Blood viscosity may affect the mechanisms of stroke and early neurological deterioration (END). We aimed to investigate the relationship between blood viscosity, stroke mechanisms, and END in patients with middle cerebral artery (MCA) infarction. Patients with symptomatic MCA atherosclerosis (≥ 50% stenosis) were recruited. Blood viscosity was compared across patients with different mechanisms of symptomatic MCA disease: in situ thrombo-occlusion (sMCA-IST), artery-to-artery embolism (sMCA-AAE), and local branch occlusion (sMCA-LBO). END was defined as four points increase in the National Institutes of Health Stroke Scale score from baseline during the first week. The association between blood viscosity and END was also evaluated. A total of 360 patients (76 with sMCA-IST, 216 with sMCA-AAE, and 68 with sMCA-LBO) were investigated. Blood viscosity was highest in patients with sMCA-IST, followed by sMCA-AAE and sMCA-LBO (*P* < 0.001). Blood viscosity was associated with END in patients with MCA disease. Low shear viscosity was associated with END in patients with sMCA- LBO (adjusted odds ratio, aOR 1.524; 95% confidence interval, CI 1.035–2.246), sMCA- IST (aOR 1.365; 95% CI 1.013–1.839), and sMCA- AAE (aOR 1.285; 95% CI 1.010–1.634). Blood viscosity was related to END in patients with stroke caused by MCA disease.

## Introduction

Atherosclerosis of the intracranial arteries which included middle cerebral artery (MCA) is usually shown on Asian patients with stroke^1,2^. Ischemic stroke caused by MCA atherosclerosis occurs through a variety of mechanism, including in situ thrombo-occlusion (IST), artery-to-artery embolism (AAE), local branch occlusion (LBO), hemodynamic impairment with linear border-zone infarction, and combined mechanisms classified by the lesion patterns^3–5^. Efforts have been made to explore the factors associated with various stroke mechanisms in MCA disease. Platelet activation, length of stenosis, and hemodynamic factors, including wall shear stress (WSS) and vulnerable plaques on high-resolution vessel wall imaging (HRVWI), are related to various stroke mechanisms in MCA disease^6–9^.

Whole blood viscosity (WBV) is the resistance of blood to flow within a vessel, which alters WSS. Therefore, WBV may influence the mechanism of stroke in patients with MCA disease. Furthermore, increased WBV is related to the prognosis of various vascular diseases^10,11^. Early neurological deterioration (END) and the prognosis of stroke caused by small vessel disease are also affected by WBV^12–14^. WBV also affects the progression of atherosclerosis, platelet aggregation, and plaque rupture that it is a likely source of influence on the mechanisms of ischemic stroke in patients with MCA disease^15^. Previous studies showed that various factors including hematocrit value, low-density lipoprotein, and fibrinogen level influence blood viscosity^16,17^.

Here, we compared differences in WBV among different stroke mechanisms in patients with symptomatic MCA atherosclerosis. The effect of WBV on END with respect to stroke mechanism was also investigated.

## Results

**Figure 1:**
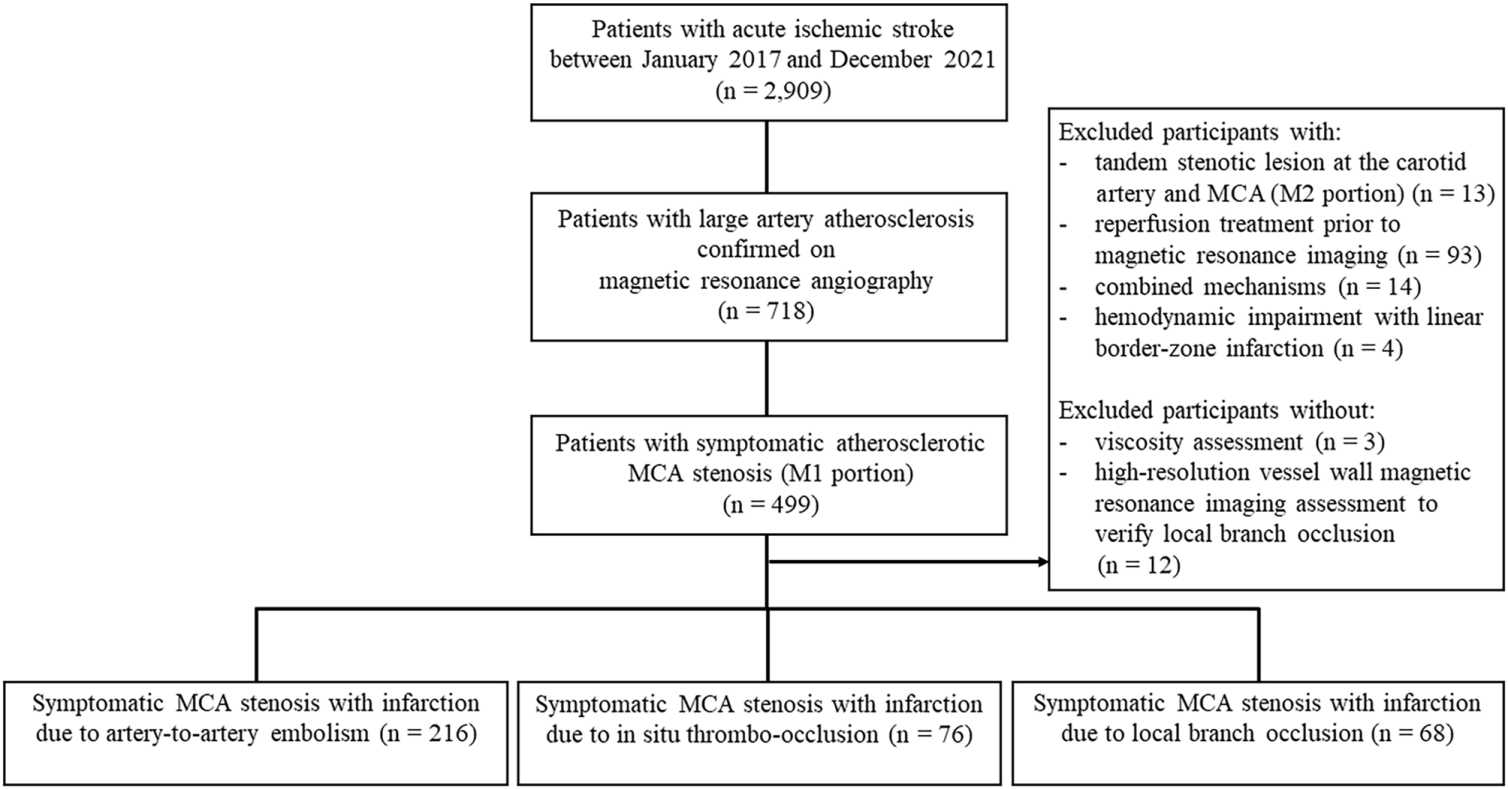
Flowchart of patient selection. *MCA* middle cerebral artery.

During the study period, 2909 patients experienced acute ischemic stroke. Among them, 718 (24.7%) were categorized as having large-artery atherosclerosis. Among them, 499 (69.4%) had a stroke in the MCA territory. After excluding these patients who had tandem stenotic lesions at the M2 portion of the MCA and carotid artery, those that did not allow WBV measurement, and those who treated with intravenous and intra-arterial thrombolysis, 360 patients with symptomatic MCA disease were included in the final analysis (Fig. 1).

The mean age of the sample was 70.4 ± 11.5 years, and 62.5% were male. Across all cases, 76 (21.1%) patients were classified as sMCA-IST, 216 (60.0%) as sMCA-AAE, and 68 (18.8%) as sMCA-LBO.

### Stroke mechanism and blood viscosity

Mean age significantly differed according to the mechanism of stroke (*P* = 0.003); patients with sMCA-LBO were older than those with sMCA-AAE and sMCA-IST. The sex ratio also differed according to the mechanism of stroke (*P* = 0.002); patients with sMCA-AAE had a higher proportion of males, whereas females were more prominent in the sMCA-LBO group. There were no significant intergroup differences in cardiovascular risk factors, previous medication, or systolic and diastolic blood pressure. The initial stroke severity was different across stroke mechanisms (*P* < 0.001); the NHISS score was higher in patients with sMCA-IST than in those with sMCA-AAE or sMCA-LBO (Table 1).

**Table 1:** Baseline characteristics according to stroke mechanism.

Characteristic	sMCA			*P*
	IST (n = 76)	AAE (n = 216)	LBO (n = 68)	
Age, y	66.6 ± 13.9	71.0 ± 10.6	72.6 ± 10.4	0.003
Male sex	48 (63.2)	147 (68.1)	30 (44.1)	0.002
Hypertension	56 (73.7)	163 (75.5)	58 (85.3)	0.183
Diabetes mellitus	28 (36.8)	77 (35.6)	26 (38.2)	0.924
Hyperlipidemia	56 (73.7)	148 (68.5)	46 (67.6)	0.659
Smoking	30 (39.5)	78 (36.1)	16 (23.5)	0.095
History of stroke	26 (34.2)	57 (26.4)	17 (25.0)	0.361
Previous medication				
Antithrombotics	28 (36.8)	80 (37.0)	32 (47.1)	0.308
Statin	22 (28.9)	91 (42.1)	26 (38.2)	0.127
Initial NIHSS score	8 (4.0–13.0)	5 (2.0–7.0)	4 (2.0–6.0)	< 0.001
Systolic blood pressure, mmHg	156.5 ± 22.1	152.7 ± 22.9	153.3 ± 24.1	0.454
Diastolic blood pressure, mmHg	85.7 ± 9.9	81.7 ± 13.8	83.0 ± 11.4	0.056
Blood laboratory findings				
White blood cell count, × 10^9^/L	9.2 ± 2.9	8.4 ± 2.8	7.4 ± 2.1	< 0.001
Hematocrit, %	43.0 ± 5.1	40.6 ± 5.3	39.9 ± 6.6	0.001
Hemoglobin, mg/dL	14.6 ± 1.7	13.8 ± 1.9	13.4 ± 2.3	0.001
Platelet count, × 10^9^/L	257.4 ± 134.3	243.5 ± 72.0	228.0 ± 66.5	0.137
Blood urea nitrogen, mg/dL	16.5 ± 7.1	18.2 ± 7.1	18.2 ± 8.1	0.187
Creatinine, mg/dL	1.0 ± 1.2	0.9 ± 0.3	0.8 ± 0.3	0.224
Total cholesterol, mg/dL	195.2 ± 63.3	183.2 ± 58.1	184.8 ± 56.1	0.307
Triglyceride, mg/dL	160.3 ± 93.7	159.8 ± 97.4	137.4 ± 64.5	0.188
High density lipoprotein, mg/dL	45.3 ± 14.8	47.0 ± 12.5	51.4 ± 12.7	0.015
Low density lipoprotein, mg/dL	120.6 ± 44.4	111.1 ± 45.7	109.1 ± 45.8	0.226
HbA1c, %	6.7 ± 1.7	6.5 ± 1.6	6.6 ± 1.5	0.539
Fasting glucose, mg/dL	122.6 ± 33.4	125.0 ± 39.1	127.9 ± 48.8	0.722
Fibrinogen, mg/dL	343.0 ± 91.9	342.3 ± 96.8	313.2 ± 97.4	0.077
Blood viscosity, centipoise				
Low shear viscosity	14.1 (12.2–16.2)	13.0 (11.6–15.7)	11.7 (11.1–13.2)	< 0.001
High shear viscosity	4.3 (3.9–4.9)	4.2 (3.8–4.9)	3.8 (3.6–4.1)	< 0.001

Values are presented as the mean ± standard deviation, number (%), or median (interquartile range). The three groups were compared using Pearson’s Chi-square tests, one-way analyses of variance, or Kruskal–Wallis tests, as appropriate.

*AAE* infarction due to artery-to-artery embolism, *IST* infarction due to in situ thrombo-occlusion, *LBO* infarction due to local branch occlusion, *MCA* middle cerebral artery, *NIHSS* National Institutes of Health Stroke Scale, *sMCA* symptomatic middle cerebral artery stenosis.

Low shear viscosity (LSV) and high shear viscosity (HSV) significantly differed according to the stroke mechanism (*P* < 0.001 and *P* < 0.001, respectively; Table 1). Compared to patients with sMCA-AAE (median [interquartile range], 13.0 [11.6–15.7] and 4.2 [3.8–4.9], respectively), LSV and HSV were higher in those with sMCA-IST (14.1 [12.2–16.2] and 4.3 [3.9–4.9], respectively) but lower in patients with sMCA-LBO (11.7 [11.1–13.2] and 3.8 [3.6–4.1], respectively; Table 1). Using sMCA-LBO status as a reference, sMCA-AAE and sMCA-IST status were independently associated with increased LSV (OR 1.186; 95% CI 1.060–1.327; *P* = 0.003 and OR 1.425; 95% CI 1.241–1.636; *P* < 0.001, respectively; Supplementary Table S1). Furthermore, sMCA-AAE and sMCA-IST status were independently associated with increased HSV (reference sMCA-LBO: OR 2.377; 95% CI 1.470–3.845; *P* < 0.001 and OR 3.854; 95% CI 2.215–6.706; *P* < 0.001, respectively; Supplementary Table S2).

A higher LSV was associated with younger age, low platelet count, low high-density lipoprotein cholesterol level, low blood urea nitrogen level, and high diastolic blood pressure, high white blood cell count, hematocrit, low-density lipoprotein cholesterol, total cholesterol, triglyceride, and fasting glucose levels (Supplementary Table S3). The factors associated with HSV were similar (Supplementary Table S4).

### Early neurological deterioration and blood viscosity

Among the enrolled patients, END was noted in 86 (23.9%). The prevalence of END differed according to stroke mechanism (*P* = 0.002); END was more frequently observed in patients with sMCA-IST (36.8%) than in those with sMCA-LBO (29.4%) or sMCA-AAE (17.6%). Patients with END had a higher LSV and HSV (*P* < 0.001 and *P* = 0.001, respectively; Table 2).

**Table 2:** Comparison of clinical and laboratory findings according to early neurological deterioration after index stroke.

	END (−) (n = 274)	END (+) (n = 86)	*P*
Demographics			
Sex, male	161 (58.8)	64 (74.4)	0.009
Age, years	70.6 ± 11.6	69.9 ± 11.4	0.658
Risk factors			
Hypertension	208 (75.9)	69 (80.2)	0.407
Diabetes mellitus	94 (34.3)	37 (43.0)	0.143
Hyperlipidemia	197 (71.9)	53 (61.6)	0.071
Smoking	91 (33.2)	33 (38.4)	0.380
History of stroke	74 (27.0)	26 (30.2)	0.582
Prior medication			
Antithrombotics	114 (41.6)	26 (30.2)	0.059
Statins	108 (39.4)	31 (36.0)	0.576
Initial NIHSS score	4 [1.0–7.0]	6 [4.0–10.3]	< 0.001
Stroke mechanism			0.002
Artery-to-artery embolism	178 (63.1)	38 (48.7)	
In situ thrombo-occlusion	48 (17.5)	20 (23.3)	
Local branch occlusion	48 (17.5)	28 (32.6)	
Systolic blood pressure, mmHg	153.3 ± 23.1	154.5 ± 22.3	0.675
Diastolic blood pressure, mmHg	82.5 ± 13.3	83.6 ± 10.8	0.445
Blood laboratory findings			
White blood cell count, × 10^9^/L	8.5 ± 2.9	8.1 ± 2.4	0.223
Hematocrit, %	40.5 ± 5.8	42.4 ± 4.7	0.007
Hemoglobin, mg/dL	13.7 ± 2.0	14.4 ± 1.6	0.002
Platelet count, × 10^9^/L	248.3 ± 94.8	228.4 ± 61.1	0.068
Blood urea nitrogen, mg/dL	18.1 ± 7.3	17.2 ± 7.3	0.309
Creatinine, mg/dL	0.9 ± 0.3	1.0 ± 1.2	0.468
Total cholesterol, mg/dL	184.6 ± 58.5	190.6 ± 60.4	0.414
Triglyceride, mg/dL	152.4 ± 83.3	166.0 ± 113.7	0.308
High-density lipoprotein, mg/dL	48.4 ± 13.6	44.7 ± 11.4	0.024
Low-density lipoprotein, mg/dL	111.1 ± 46.3	118.0 ± 42.8	0.219
HbA1c, %	6.5 ± 1.5	6.7 ± 1.6	0.164
Fasting glucose, mg/dL	123.3 ± 39.9	130.5 ± 39.7	0.146
Fibrinogen, mg/dL	338.9 ± 97.9	330.7 ± 91.5	0.487
Blood viscosity, centipoise			
Low shear viscosity	12.4 [11.3–15.5]	13.5 [12.2–16.5]	< 0.001
High shear viscosity	4.0 [3.7–4.8]	4.3 [4.0–5.0]	0.001

Data are presented as n (%), mean ± standard deviation, or median [interquartile range].

*END* early neurological deterioration, *NIHSS* National Institutes of Health Stroke Scale.

After adjustment, multivariable logistic regression analysis showed that END was independently related to increased LSV (OR 1.319; 95% CI 1.150–1.513; *P* < 0.001), higher initial NIHSS score (OR 1.139; 95% CI 1.076–1.206; *P* < 0.001), and sMCA-IST (OR 4.160; 95% CI 1.985–8.715; *P* < 0.001; Table 3, Supplementary Table S5). In another model, END was independently related to an increased HSV (OR 1.685; 95% CI 1.011–2.810; *P* = 0.045; Supplementary Tables S5 and S6).

**Table 3:** Multivariable analysis for early neurological deterioration after ischemic stroke with the following dependent variables.

Variables	*Odds ratio (95% CI)	*P*
Sex, male	1.510 (0.761–2.995)	0.238
Age, years	1.007 (0.980–1.034)	0.629
History of hyperlipidemia	0.620 (0.333–1.153)	0.131
Prior antithrombotics use	0.912 (0.474–1.754)	0.783
Initial NIHSS score	1.139 (1.076–1.206)	< 0.001
Low shear viscosity	1.319 (1.150–1.513)	< 0.001
Stroke mechanism		
Artery-to-artery embolism	1 (served as reference)	
In situ thrombo-occlusion	4.160 (1.985–8.715)	< 0.001
Local branch occlusion	1.671 (0.831–3.358)	0.149
Hemoglobin	0.998 (0.832–1.197)	0.982
Platelet count	0.998 (0.994–1.002)	0.346
High-density lipoprotein	0.991 (0.968–1.014)	0.436

Data are shown as odds ratio (95% confidence interval).

*CI* confidence interval, *NIHSS* National Institutes of Health Stroke Scale.

*Adjusted for sex, age, history of hyperlipidemia, prior antithrombotic use, initial NIHSS score, low shear viscosity, stroke mechanism, hemoglobin, platelet count, and high-density lipoprotein.

### Early neurological deterioration and blood viscosity in stroke subtypes

In patients with sMCA-IST, LSV was significantly higher in END group compared to non-END group (14.3 [13.7–18.6] versus 13.7 [11.7–16.1]; *P* = 0.009; Supplementary Table S7). High-density lipoprotein (adjusted OR 0.912; 95% CI 0.855–0.973) and LSV (adjusted OR 1.365; 95% CI 1.013–1.839) were related to END in patients with sMCA-IST (Supplementary Table S8). In patients with sMCA-AAE, compared to non-END group (12.7 [11.5–15.6] and 4.1 [3.8–4.9], respectively), LSV and HSV were significantly higher in END group (13.5 [12.6–16.4] and 4.3 [4.0–4.9], respectively; Supplementary Table S9). In patients with sMCA-AAE, a history of hypertension (adjusted OR 4.922; 95% CI 1.388–17.460), diabetes mellitus (adjusted OR 5.423; 95% CI 1.759–16.715), initial NIHSS score (adjusted OR 1.164; 95% CI 1.072–1.265), blood urea nitrogen (adjusted OR 0.921; 95% CI 0.854–0.995), and LSV (adjusted OR 1.285; 95% CI 1.010–1.634) were related to END (Supplementary Table S10). In patients with sMCA-LBO, LSV and HSV were significantly higher in END group (13.0 [11.4–17.1] and 4.0 [3.7–5.1], respectively) compared to non-END group (11.6 [10.3–13.0] and 3.8 [3.5–4.0], respectively; Supplementary Table S11). In patients with sMCA-LBO, LSV (adjusted OR 1.524; 95% CI 1.035–2.246) was related to END (Supplementary Table S12).

## Discussion

In the present study, the LSV and HSV were significantly different among patients with sMCA-IST, sMCA-AAE, and sMCA-LBO. LSV and HSV were highest in patients with sMCA-IST, followed by patients with sMCA-AAE and sMCA-LBO. Previous study suggested that LSV was higher in stroke due to small artery occlusion than stroke due to large artery atherosclerosis and impairs microvascular tissue perfusion^13^. Because patho-mechanisms and END rates were different between stroke due to small artery occlusion and sMCA-LBO^18,19^, sMCA-IST group showed higher LSV than sMCA-LBO group and high LSV was more associated with END in sMCA-LBO rather than END in sMCA-IST and sMCA-AAE in our study. High blood viscosity has been shown to result in high WSS^20^. Previous studies have documented that high WSS provokes plaque rupture which can lead to IST^21,22^. Furthermore, high or alternating WSS causes shear-induced platelet activation, which may cause thrombus formation, distal embolization, and ischemic stroke (artery-to-artery embolization)^23,24^. Furthermore, high blood viscosity itself provokes blood cell aggregation, reducing blood flow and ultimately leading to thrombosis^25^.

In this study, patients with END had higher LSV and HSV than did those without END. Furthermore, association between END and LSV were highest in patients with sMCA-LBO (adjusted OR 1.524; 95% CI 1.035–2.246), followed by patients with sMCA-IST (adjusted OR 1.365; 95% CI 1.013–1.839) and sMCA-AAE (adjusted OR 1.285; 95% CI 1.010–1.634). END was frequently observed in patients with large-artery atherosclerosis. Among the various mechanisms of END, stroke progression is the most common cause of END in large-artery atherosclerosis^26^. Stroke progression is caused by reduced blood flow, poor collateral blood flow, clot propagation, and vasogenic edema^27,28^. In low shear rate, LSV contribute to develop rouleaux structure (red blood cell aggregate) which may be more closely related to perfusion of perforating artery than large artery and clot propagation^13,29^. In cases of sMCA-LBO, increased LSV may affect the flow to the perforators by increasing the resistance to microvascular tissue perfusion^30^. In patients with sMCA-IST, increased blood viscosity may be related to decreased tissue perfusion distal to the occlusion site^31^. Furthermore, high blood viscosity is associated with poor collateralization in coronary artery occlusion^32^. Elevated blood viscosity in patients with sMCA-AAE may be associated with clot propagation and recurrent embolism from the high WSS area^33^. Various mechanisms affected by the increased blood viscosity may help explain the broad association between END and WBV in MCA disease. Interestingly, LSV appears to be a more important factor than HSV for END. This may be at least partially explained by the fact that LSV measured at a low shear rate is more important for tissue perfusion and thrombus propagation, which are mechanisms of END, than HSV measured at a high shear rate^29^. In addition, patients with sMCA-LBO had older age, higher proportion of female, higher high-density lipoprotein than other subgroups. Patients with sMCA-IST had younger age, higher white blood cell count, and higher hemoglobin than other subgroups. And, patients with sMCA-AAE had higher proportion of male than other subgroups. These conditions in each subgroup help that higher viscosity can be dangerous for END.

Our study has several limitations. First, selection bias stemming from patient consent and the cross-sectional design cannot be excluded, although we tried to consecutively enroll all pertinent patients. Second, we analyzed the WBV acquired within 24 h of admission. Therefore, we could not examine serial changes in WBV during the course of the stroke. Furthermore, patients with acute ischemic stroke aroused by large-artery atherosclerosis were treated with hydration during the hyperacute period. Because blood viscosity can be usually affected by hydration therapy for fluid infusion, follow-up assessments of blood viscosity would have been ideal. Third, patients with acute ischemic stroke aroused by LBO and AAE may also have less than 50% stenosis of the MCA. Because patients with stroke were evaluated using HRVWI, if more than 50% relevant stenosis in the MCA was confirmed on TOF MRA, selection bias caused by the inclusion of patients cannot be excluded. Fourth, composition and vulnerability of the plaque, embolus size, and each stroke mechanism were not confirmed pathologically. Fifth, results of a recent detailed time study on viscosity and other rheological blood measurements have shown significant changes in the viscosity measurements taking place within the first 24 h with a recommended time window for the measurements of 4 h^34^. In our study, this extended time window has caused a bias in the measurements. However, we conducted blood collection and viscosity measurements per our hospital protocol and association between END and time window between time on viscosity measurements and time on admission at our hospital also did not show (OR 1.013; 95% CI 0.969–1.058) and time window randomly scattered. Finally, the relationship between WBV and other additional features, such as flow velocity and WSS, were not assessed.

In conclusion, present study first reported a relation between blood viscosity, stroke mechanism, and END in patients with MCA atherosclerosis. Acute ischemic stroke in the MCA territory aroused by IST is associated with the highest blood viscosity. Low shear viscosity is more closely related to END in patients with acute ischemic stroke due to local branch occlusion with MCA atherosclerosis.

## Methods

### Participants

Between January 2017 and December 2021, prospectively assembled patients with ischemic stroke and atherosclerotic stenosis of the MCA confirmed using magnetic resonance imaging (MRI) and magnetic resonance angiography (MRA) were retrospectively analyzed. Symptomatic atherosclerotic MCA stenosis was defined as patients with acute ischemic stroke (within 7 days of stroke onset) seen on diffusion-weighted imaging (DWI) who had MCA stenosis of 50% or greater.

We eliminated patients who had (1) tandem stenotic lesions (significant stenosis or occlusion) at the carotid artery and M2 portion of the MCA, (2) nonatherosclerotic intracranial stenosis such as Moyamoya disease, vasculitis, or dissection, or (3) potential embolic source (e.g., coagulopathy or cardioembolic source). We also excluded patients who (4) underwent reperfusion treatment, including thrombolysis and thrombectomy, prior to the assessment of WBV, or (5) had poor imaging quality. This study was approved by the Institutional Review Board of the Kyung Hee University Hospital (no. 2009-12-301), which waived the requirement for informed consent due to retrospective nature of study and minimal risk of data collection to patients.

### Clinical data

Baseline characteristics, cardiovascular risk factors, and concomitant medications were investigated. Stroke severity was measured using the National Institutes of Health Stroke Scale (NIHSS) score, with initial evaluation conducted upon arrival to the emergency department (baseline) and daily reevaluations by experienced stroke neurologists during the full length of hospitalization. END was defined as four points increase in the NIHSS score from baseline during the first week of hospitalization^27,35^.

### Stroke mechanism and imaging

On the first day of admission, patients with acute ischemic stroke and stenosis of the MCA underwent 3T MRI and MRA (Intera; Philips Medical Systems, Best, Netherlands). If more than 50% relevant stenosis of the MCA was observed on time-of-flight (TOF) MRA, those additionally evaluated HRVWI (Supplementary Method).

The mechanism of stroke aroused by atherosclerotic stenosis of the MCA was categorized by the topographic appearance on DWI and determined by consensus. Patients with a small (diameter < 2 cm) single subcortical lesion that is accompanied by a positional relation between the location of plaque and the perforating vessels of MCA on HRVWI were categorized as having LBO. Patients with small multiple cortical lesions were classified as having AAE. Patients with large cortical and subcortical territorial infarction caused by MCA occlusion, except those who had MCA occlusion due to AAE from the proximal arteries or other combined mechanisms, were categorized as having IST. Linear (chain-like) internal border zone or wedge-shaped external border-zone infarction were classified as hemodynamic impairment with linear border-zone infarction. Lastly, small multiple cortical and subcortical lesions were categorized as mixed mechanisms^36^.

As it is difficult to determine causality in patients with infarctions from combined mechanisms and given that patients with hemodynamic impairment from linear border-zone infarctions are rare, those with these mechanisms were not entered in the final analysis. Among patients with symptomatic MCA disease, data from those with stroke aroused by LBO, AAE, and IST, were retained for analysis.

### Whole blood viscosity measurement

A scanning capillary tube viscometer (BVD-PR01, Bio-Visco Inc., Jeonju, Korea) was used to evaluate the WBV of each enrolled patient. Blood samples containing 6 mL of whole blood, acquired on the first day of admission, were assembled in a vacutainer containing ethylenediaminetetraacetic acid anticoagulant and kept in a refrigerator at 4 °C until measurement. All assessments were conducted within 24 h of collection. WBV was assessed over a wide range of shear rates, ranging from 1 to 1000 s^−1^. WBV assessed at a high shear rate of 300 s^−1^ is termed to as HSV, whereas that assessed at a low shear rate of 5 s^−1^ is termed to as LSV.

### Statistical methods

Baseline characteristics, cardiovascular risk factors, concomitant medications, and WBV (LSV and HSV) were compared among patients with symptomatic MCA disease with LBO (sMCA-LBO), AAE (sMCA-AAE), and IST (sMCA-IST). We performed Kolmogorov–Smirnov test to verify normal distribution of LSV and HSV and confirmed that LSV and HSV does not follow a normal distribution. Kruskal–Wallis tests, Pearson’s Chi-square tests, and one-way analyses of variance were used as suitable. Multivariable logistic regression was performed to evaluate the independent relationship between stroke mechanism and blood viscosity. Also, baseline characteristics, cardiovascular risk factors, concomitant medications, and WBV (LSV and HSV) were compared between END group and non-END group. Spearman rank-correlation analyses were performed to examine the relationship between WBV and initial stroke severity (NIHSS score) and other blood laboratory findings. Next, we analyzed the association between END and WBV using binary logistic regression. Multivariable logistic regression analysis involved age, sex, and all of the candidate factors with values of *P* < 0.1 in the preliminary univariable analyses.

We performed a subgroup analysis according to each stroke mechanism. Odds ratios (ORs) were estimated with 95% confidence intervals (CIs). *P* values less than 0.05 were typically referred to be statistically significant. Analyses were conducted using SPSS 22.0 for Windows (IBM Corp., Armonk, NY, USA).

## Supplementary Information

Supplementary Information.

## Data Availability

The data set supporting the results of our study are available from the corresponding author upon reasonable request, who uses the full data set and takes responsibility for its integrity and analysis.
