# Supplementary material for: Blood viscosity associated with stroke mechanism and early neurological deterioration in middle cerebral artery atherosclerosis
Source: Sci Rep. 2023 Jun 9;13:9384. doi: 10.1038/s41598-023-36633-z (PMC10256783; doi:10.1038/s41598-023-36633-z)
Supplement: Supplementary file 1 — Supplementary Information. [file 41598_2023_36633_MOESM1_ESM.docx]

**Supplementary Method**

**Imaging protocol**

Patients with symptomatic middle cerebral artery (MCA) disease underwent 3-T magnetic resonance imaging and magnetic resonance angiography (MRA) (Intera; Philips Medical Systems, Best, Netherlands) on the first day of admission to the stroke unit including the following sequences: diffusion-weighted imaging, T1- and T2-weighted imaging, fluid attenuated inversion recovery imaging, susceptibility-weighted imaging, time-of-flight (TOF) MRA (intracranial), and contrast-enhanced MRA (extracranial).

If patient with local branch occlusion was suspected on TOF MRA, the patients also underwent high-resolution vessel wall magnetic resonance imaging to confirm spatial relationship between atherosclerotic plaque and orifice of perforator, which was performed using a using a 3-T Philips scanner (Philips Healthcare, Eindhoven, Netherlands) with a standard 16-channel neurovascular coil. The sequences included TOF MRA, maximum-intensity projection, T1-weighted imaging, proton-density black blood image (PD-BBI), and contrast-enhanced T1-weighted imaging with turbo/fast spin-echo sequences. T1-weighted spin echo was obtained using the following parameters (in-plane resolution=0.5×0.5 mm, slice thickness/spacing=0.5/0.5 mm, repetition time (TR)/echo time (TE)=650/16 ms, field-of-view (FOV)=180×180 mm, matrix=360×360, time=7 min 20 s), voxel size=0.35×0.35×0.5 mm^3^. PD-BBI was obtained using the following parameters along the MCA axis: TR/TE=2,000/30 ms, FOV=180×180 mm, matrix=360×360, slice thickness/spacing=0.5/0.5 mm. Contrast-enhanced T1-weighted imaging after intravenous gadolinium administration (Gadovist; Bayer Schering Pharma, Berlin, Germany) was obtained. A pre-regional 80-mm-thick saturation pulse was used to saturate the incoming arterial flow.

**Supplementary Table S1.** Multivariable analysis for low shear viscosity according to stroke mechanisms

| Variables | Odds ratio (95% CI) | *P* |
| --- | --- | --- |
| Stroke mechanism |  |  |
| Artery-to-artery embolism | 1.186 (1.060–1.327) | 0.003 |
| In situ thrombo-occlusion | 1.425 (1.241–1.635) | <0.001 |
| Local branch occlusion | 1 (served as reference) |  |

Data are shown as odds ratio (95% confidence interval).

CI, confidence interval

**Supplementary Table S2.** Multivariable analysis for high shear viscosity according to stroke mechanisms

| Variables | Odds ratio (95% CI) | *P* |
| --- | --- | --- |
| Stroke mechanism |  |  |
| Artery-to-artery embolism | 2.377 (1.470–3.845) | <0.001 |
| In situ thrombo-occlusion | 3.854 (2.215–6.706) | <0.001 |
| Local branch occlusion | 1 (served as reference) |  |

Data are shown as odds ratio (95% confidence interval).

CI, confidence interval

**Supplementary Table S3.** Correlation of low shear viscosity with clinical and laboratory findings.

| Variable | *Spearman’s Rho* | *P* |
| --- | --- | --- |
| Age, y | −0.225 | <0.001 |
| Initial NIHSS score | 0.007 | 0.896 |
| Systolic blood pressure, mmHg  Diastolic blood pressure, mmHg | −0.053  0.151 | 0.314  0.004 |
| Blood laboratory findings  White blood cell count, x10^9^/L  Hematocrit, %  Hemoglobin, mg/dL  Platelet count, x10^9^/L  Blood urea nitrogen, mg/dL  Creatinine, mg/dL  Total cholesterol, mg/dL  Triglyceride, mg/dL  High-density lipoprotein, mg/dL  Low-density lipoprotein, mg/dL  HbA1c, %  Fasting glucose, mg/dL  Fibrinogen, mg/dL | 0.137  0.618  0.644  −0.111  −0.196  0.120  0.232  0.362  −0.227  0.253  −0.020  0.112  0.058 | 0.009  <0.001  <0.001  0.035  <0.001  0.023  <0.001  <0.001  <0.001  <0.001  0.708  0.033  0.275 |

NIHSS, National Institutes of Health Stroke Scale.

**Supplementary Table S4.** Correlation of high shear viscosity with clinical and laboratory findings.

| Variable | *Spearman’s Rho* | *P* |
| --- | --- | --- |
| Age, y | −0.239 | <0.001 |
| Initial NIHSS score | −0.010 | 0.853 |
| Systolic blood pressure, mmHg  Diastolic blood pressure, mmHg | −0.066  0.140 | 0.214  0.008 |
| Blood laboratory findings  White blood cell count, x10^9^/L  Hematocrit, %  Hemoglobin, mg/dL  Platelet count, x10^9^/L  Blood urea nitrogen, mg/dL  Creatinine, mg/dL  Total cholesterol, mg/dL  Triglyceride, mg/dL  High-density lipoprotein, mg/dL  Low-density lipoprotein, mg/dL  HbA1c, %  Fasting glucose, mg/dL  Fibrinogen, mg/dL | 0.174  0.640  0.663  −0.096  −0.167  0.132  0.220  0.377  −0.195  0.245  −0.003  0.117  0.061 | 0.001  <0.001  <0.001  0.068  0.001  0.012  <0.001  <0.001  <0.001  <0.001  0.950  0.026  0.252 |

NIHSS, National Institutes of Health Stroke Scale.

**Supplementary Table S5.** Associated factors for early neurological deterioration after ischemic stroke.

| Characteristic | Odds ratio (95% CI) | *P* |
| --- | --- | --- |
| Demographics |  |  |
| Sex, male | 2.042 (1.189–3.507) | 0.010 |
| Age, years | 0.995 (0.975–1.016) | 0.657 |
| Risk factors |  |  |
| Hypertension | 1.288 (0.708–2.343) | 0.407 |
| Diabetes mellitus | 1.446 (0.882–2.371) | 0.144 |
| Hyperlipidemia | 0.628 (0.378–1.044) | 0.073 |
| Smoking  History of stroke | 1.252 (0.758–2.069)  1.171 (0.688–1.993) | 0.380  0.560 |
| Prior medication |  |  |
| Antithrombotics | 0.608 (0.362–1.022) | 0.060 |
| Statins | 0.866 (0.524–1.432) | 0.576 |
| Initial NIHSS score | 1.122 (1.071–1.176) | <0.001 |
| Stroke mechanism |  |  |
| Artery-to-artery embolism | 1 (Reference) |  |
| In situ thrombo-occlusion | 2.732 (1.525–4.895) | 0.001 |
| Local branch occlusion | 1.952 (1.041–3.658) | 0.037 |
| Systolic blood pressure, mmHg | 1.002 (0.992–1.013) | 0.674 |
| Diastolic blood pressure, mmHg | 1.007 (0.988–1.026) | 0.491 |
| Blood laboratory findings |  |  |
| White blood cell count, x10^9^/L | 0.945 (0.862–1.035) | 0.223 |
| Hematocrit, % | 1.069 (1.018–1.123) | 0.007 |
| Hemoglobin, mg/dL | 1.252 (1.082–1.449) | 0.002 |
| Platelet count, x10^9^/L | 0.997 (0.993–1.000) | 0.065 |
| Blood urea nitrogen, mg/dL | 0.982 (0.948–1.017) | 0.309 |
| Creatinine, mg/dL | 1.209 (0.863–1.695) | 0.269 |
| Total cholesterol, mg/dL | 1.002 (0.998–1.006) | 0.413 |
| Triglyceride, mg/dL | 1.002 (0.999–1.004) | 0.232 |
| High-density lipoprotein, mg/dL | 0.977 (0.958–0.997) | 0.025 |
| Low-density lipoprotein, mg/dL | 1.003 (0.998–1.008) | 0.221 |
| HbA1c, % | 1.107 (0.958–1.279) | 0.167 |
| Fasting glucose, mg/dL | 1.004 (0.999–1.010) | 0.147 |
| Fibrinogen, mg/dL | 0.999 (0.996–1.002) | 0.486 |
| Blood viscosity, centipoise |  |  |
| Low shear viscosity | 1.308 (1.183–1.447) | <0.001 |
| High shear viscosity | 1.911 (1.344–2.717) | <0.001 |

Data are shown as odds ratio (95% confidence interval).

CI, confidence interval; NIHSS, National Institutes of Health Stroke Scale.

**Supplementary Table S6.** Multivariable analysis for early neurological deterioration after ischemic stroke with following dependent variables.

| Variables | ^*^Odds ratio (95% CI) | *P* |
| --- | --- | --- |
| Sex, male | 1.522 (0.775–2.986) | 0.222 |
| Age, years | 1.007 (0.981–1.034) | 0.599 |
| History of hyperlipidemia | 0.622 (0.340–1.140) | 0.124 |
| Prior antithrombotics use | 0.775 (0.411–1.461) | 0.312 |
| Initial NIHSS score | 1.134 (1.074–1.199) | <0.001 |
| High shear viscosity | 1.685 (1.011–2.810) | 0.045 |
| Stroke mechanism |  |  |
| Artery-to-artery embolism | 1 (Reference) |  |
| In situ thrombo-occlusion | 3.679 (1.770–7.645) | <0.001 |
| Local branch occlusion | 1.823 (0.924–3.597) | 0.083 |
| Hemoglobin | 1.156 (0.891–1.306) | 0.439 |
| Platelet count | 0.998 (0.994–1.002) | 0.276 |
| High-density lipoprotein | 0.985 (0.962–1.008) | 0.194 |

Data are shown as odds ratio (95% confidence interval).

CI, confidence interval; NIHSS, National Institutes of Health Stroke Scale.

*: Adjusted for sex, age, history of hyperlipidemia, prior antithrombotics use, initial NIHSS score, high shear viscosity, stroke mechanism, hemoglobin, platelet count, and high-density lipoprotein.

**Supplementary Table S7.** Comparison of clinical and laboratory findings according to early neurological deterioration after index stroke caused by in situ thrombo-occlusion

|  | END (-) (n = 48) | END (+) (n = 28) | *P* |
| --- | --- | --- | --- |
| Demographics |  |  |  |
| Sex, male | 27 (56.3) | 21 (75.0) | 0.102 |
| Age, years | 66.9 ± 13.7 | 66.0 ± 14.5 | 0.787 |
| Risk factors |  |  |  |
| Hypertension | 36 (75.0) | 20 (71.4) | 0.733 |
| Diabetes mellitus | 16 (33.3) | 12 (42.9) | 0.406 |
| Hyperlipidemia | 37 (77.1) | 19 (67.9) | 0.378 |
| Smoking | 21 (43.8) | 9 (32.1) | 0.318 |
| History of stroke | 15 (31.3) | 11 (39.3) | 0.476 |
| Prior medication |  |  |  |
| Antithrombotics | 14 (29.2) | 14 (50.0) | 0.069 |
| Statins | 12 (25.0) | 10 (35.7) | 0.320 |
| Initial NIHSS score | 7.5 [1.3 – 11.0] | 8.5 [5.0 – 17.0] | 0.036 |
| Systolic blood pressure, mmHg | 161.7 ± 21.5 | 147.5 ± 20.3 | 0.006 |
| Diastolic blood pressure, mmHg | 87.2 ± 10.2 | 83.1 ± 8.8 | 0.080 |
| Blood laboratory findings |  |  |  |
| White blood cell count, x10^9^/L | 9.2 ± 3.0 | 9.3 ± 2.8 | 0.917 |
| Hematocrit, % | 42.9 ± 4.9 | 43.1 ± 5.6 | 0.861 |
| Hemoglobin, mg/dL | 14.4 ± 1.6 | 14.8 ± 1.9 | 0.342 |
| Platelet count, x10^9^/L | 266.8 ± 163.4 | 241.2 ± 56.5 | 0.426 |
| Blood urea nitrogen, mg/dL | 17.2 ± 6.6 | 15.3 ± 7.8 | 0.244 |
| Creatinine, mg/dL | 0.8 ± 0.3 | 1.3 ± 2.0 | 0.247 |
| Total cholesterol, mg/dL | 205.6 ± 47.7 | 177.4 ± 81.5 | 0.103 |
| Triglyceride, mg/dL | 159.5 ± 84.8 | 161.5 ± 108.9 | 0.931 |
| High-density lipoprotein, mg/dL | 50.4 ± 15.4 | 36.4 ± 8.2 | <0.001 |
| Low-density lipoprotein, mg/dL | 125.2 ± 32.4 | 112.8 ± 59.5 | 0.316 |
| HbA1c, % | 6.7 ± 1.7 | 6.7 ± 1.6 | 0.989 |
| Fasting glucose, mg/dL | 122.0 ± 35.1 | 123.5 ± 30.8 | 0.852 |
| Fibrinogen, mg/dL | 338.2 ± 94.0 | 351.4 ± 89.2 | 0.550 |
| Blood viscosity, centipoise |  |  |  |
| Low shear viscosity | 13.7 [11.7 – 16.1] | 14.3 [13.7 – 18.6] | 0.009 |
| High shear viscosity | 4.3 [3.8 – 4.8] | 4.3 [4.1 – 5.6] | 0.131 |

Data are presented as n (%), mean ± standard deviation, or median [interquartile range].

END, early neurological deterioration; NIHSS, National Institutes of Health Stroke Scale

**Supplementary Table S8.** Associated factors for early neurological deterioration after ischemic stroke caused by in situ thrombo-occlusion

| Characteristic | Crude OR (95% CI) | Adjusted OR (95% CI)^a^ | Adjusted OR (95% CI)^b^ |
| --- | --- | --- | --- |
| Demographics |  |  |  |
| Sex, male | 2.333 (0.835–6.523) |  |  |
| Age, years | 0.995 (0.962–1.029) |  |  |
| Risk factors |  |  |  |
| Hypertension | 0.833 (0.292–2.378) |  |  |
| Diabetes mellitus | 1.500 (0.575–3.915) |  |  |
| Hyperlipidemia | 0.628 (0.222–1.776) |  |  |
| Smoking | 0.609 (0.229–1.618) |  |  |
| History of stroke | 1.424 (0.538–3.768) |  | 0.938 (0.222–3.952) |
| Prior medication |  |  |  |
| Antithrombotics | 2.429 (0.923–6.391)^†^ | 0.179 (0.018–1.732) |  |
| Statins | 1.667 (0.606–4.586) |  |  |
| Initial NIHSS score | 1.103 (1.019–1.194)^*^ | 1.088 (0.969–1.222) | 1.085 (0.976–1.206) |
| Systolic blood pressure, mmHg | 0.969 (0.945–0.992)^*^ | 0.988 (0.953–1.025) | 0.985 (0.951–1.020) |
| Diastolic blood pressure, mmHg | 0.957 (0.910–1.006)^†^ | 0.980 (0.912–1.054) | 0.977 (0.908–1.051) |
| Blood laboratory findings |  |  |  |
| White blood cell count, x10^9^/L | 1.009 (0.858–1.187) |  |  |
| Hematocrit, % | 1.008 (0.921–1.104) |  |  |
| Hemoglobin, mg/dL | 1.146 (0.867–1.516) |  |  |
| Platelet count, x10^9^/L | 0.998 (0.994–1.003) |  |  |
| Blood urea nitrogen, mg/dL | 0.957 (0.889–1.030) |  |  |
| Creatinine, mg/dL | 1.394 (0.822–2.366) |  |  |
| Total cholesterol, mg/dL | 0.992 (0.984–1.001)^†^ | 1.001 (0.990–1.011) | 0.999 (0.988–1.010) |
| Triglyceride, mg/dL | 1.000 (0.995–1.005) |  |  |
| High-density lipoprotein, mg/dL | 0.904 (0.854–0.957)^†^ | 0.904 (0.845–0.967)^*^ | 0.912 (0.855–0.973)^*^ |
| Low-density lipoprotein, mg/dL | 0.993 (0.982–1.005) |  |  |
| HbA1c, % | 0.998 (0.751–1.326) |  |  |
| Fasting glucose, mg/dL | 1.001 (0.987–1.015) |  |  |
| Fibrinogen, mg/dL | 1.002 (0.997–1.007) |  |  |
| Blood viscosity, centipoise |  |  |  |
| Low shear viscosity | 1.384 (1.116–1.716)^*^ | 1.365 (1.013–1.839)^*^ | 1.336 (1.006–1.775)^*^ |
| High shear viscosity | 1.746 (0.935–3.260)^†^ |  |  |

Data are shown as odds ratio (95% confidence interval).

CI, confidence interval; NIHSS, National Institutes of Health Stroke Scale; OR, odds ratio.

^*^p < 0.05, ^†^p < 0.1

^a^Adjusted for prior antithrombotics use, initial NIHSS score, systolic blood pressure, diastolic blood pressure, total cholesterol, high-density lipoprotein, and low shear viscosity.

^b^Adjusted for history of stroke, initial NIHSS score, systolic blood pressure, diastolic blood pressure, total cholesterol, high-density lipoprotein, and low shear viscosity.

**Supplementary Table S9.** Comparison of clinical and laboratory findings according to early neurological deterioration after index stroke caused by artery-to-artery embolism

|  | END (-) (n = 178) | END (+) (n = 38) | *P* |
| --- | --- | --- | --- |
| Demographics |  |  |  |
| Sex, male | 120 (67.4) | 27 (71.1) | 0.662 |
| Age, years | 70.9 ± 11.0 | 71.8 ± 8.7 | 0.574 |
| Risk factors |  |  |  |
| Hypertension | 130 (73.0) | 33 (86.8) | 0.096 |
| Diabetes mellitus | 58 (32.6) | 19 (50.0) | 0.042 |
| Hyperlipidemia | 124 (69.7) | 24 (63.2) | 0.433 |
| Smoking | 60 (33.7) | 18 (47.4) | 0.111 |
| History of stroke | 50 (28.1) | 7 (18.4) | 0.220 |
| Prior medication |  |  |  |
| Antithrombotics | 72 (40.4) | 8 (21.1) | 0.025 |
| Statins | 72 (40.4) | 19 (50.0) | 0.279 |
| Initial NIHSS score | 4 [1.0 – 6.0] | 7 [4.8 – 11.0] | <0.001 |
| Systolic blood pressure, mmHg | 151.0 ± 22.7 | 160.3 ± 22.5 | 0.024 |
| Diastolic blood pressure, mmHg | 80.9 ± 13.9 | 85.2 ± 13.3 | 0.083 |
| Blood laboratory findings |  |  |  |
| White blood cell count, x10^9^/L | 8.5 ± 3.0 | 8.0 ± 2.0 | 0.162 |
| Hematocrit, % | 40.3 ± 5.5 | 41.9 ± 4.2 | 0.085 |
| Hemoglobin, mg/dL | 13.7 ± 1.9 | 14.3 ± 1.5 | 0.065 |
| Platelet count, x10^9^/L | 247.2 ± 71.5 | 226.2 ± 72.7 | 0.102 |
| Blood urea nitrogen, mg/dL | 18.6 ± 7.4 | 16.3 ± 5.3 | 0.071 |
| Creatinine, mg/dL | 0.9 ± 0.3 | 0.9 ± 0.2 | 0.088 |
| Total cholesterol, mg/dL | 180.0 ± 59.3 | 198.2 ± 50.5 | 0.080 |
| Triglyceride, mg/dL | 155.7 ± 87.5 | 179.0 ± 134.3 | 0.312 |
| High-density lipoprotein, mg/dL | 46.9 ± 12.6 | 47.8 ± 11.6 | 0.658 |
| Low-density lipoprotein, mg/dL | 108.8 ± 47.5 | 121.6 ± 34.9 | 0.120 |
| HbA1c, % | 6.4 ± 1.5 | 6.8 ± 1.6 | 0.097 |
| Fasting glucose, mg/dL | 121.6 ± 36.8 | 141.0 ± 45.5 | 0.017 |
| Fibrinogen, mg/dL | 345.3 ± 100.2 | 328.6 ± 78.4 | 0.336 |
| Blood viscosity, centipoise |  |  |  |
| Low shear viscosity | 12.7 [11.5 – 15.6] | 13.5 [12.6 – 16.4] | 0.002 |
| High shear viscosity | 4.1 [3.8 – 4.9] | 4.3 [4.0 – 4.9] | 0.031 |

Data are presented as n (%), mean ± standard deviation, or median [interquartile range].

END, early neurological deterioration; NIHSS, National Institutes of Health Stroke Scale

**Supplementary Table S10.** Associated factors for early neurological deterioration after ischemic stroke caused by artery-to-artery embolism.

| Characteristic | Crude OR (95% CI) | Adjusted OR (95% CI)^a^ | | Adjusted OR (95% CI)^b^ |
| --- | --- | --- | --- | --- |
| Demographics |  |  |  | |
| Sex, male | 1.186 (0.550–2.557) |  |  | |
| Age, years | 1.008 (0.975–1.043) |  |  | |
| Risk factors |  |  |  | |
| Hypertension | 2.437 (0.899–6.605)^†^ | 4.922 (1.388–17.460)^*^ | 4.147 (1.199–14.350)^*^ | |
| Diabetes mellitus | 2.069 (1.018–4.204)^*^ | 5.423 (1.759–16.715)^*^ | 5.137 (1.688–15.634)^*^ | |
| Hyperlipidemia | 0.747 (0.359–1.553) |  |  | |
| Smoking | 1.770 (0.871–3.595) |  |  | |
| History of stroke | 0.578 (0.239-1.398) |  | 1.266 (0.377–4.246) | |
| Prior medication |  |  |  | |
| Antithrombotics | 0.393 (0.170–0.905)^*^ | 0.491 (0.152–1.588) |  | |
| Statins | 1.472 (0.729–2.973) |  |  | |
| Initial NIHSS score | 1.155 (1.077–1.239)^*^ | 1.164 (1.072–1.265)^*^ | 1.177 (1.081–1.281)^*^ | |
| Systolic blood pressure, mmHg | 1.018 (1.002–1.035)^*^ | 1.022 (0.997–1.047)^†^ | 1.025 (0.987–1.053)^†^ | |
| Diastolic blood pressure, mmHg | 1.023 (0.997–1.051)^†^ | 0.985 (0.947–1.025) | 0.989 (0.951–1.028) | |
| Blood laboratory findings |  |  |  | |
| White blood cell count, x10^9^/L | 0.927 (0.809–1.061) |  |  | |
| Hematocrit, % | 1.068 (0.991–1.152) |  |  | |
| Hemoglobin, mg/dL | 1.226 (0.987–1.523)^†^ | 0.991 (0.715–1.373) | 1.016 (0.720–1.434) | |
| Platelet count, x10^9^/L | 0.995 (0.990–1.001) |  |  | |
| Blood urea nitrogen, mg/dL | 0.948 (0.893–1.005)^†^ | 0.921 (0.854–0.995)^*^ | 0.925 (0.857–0.999)^*^ | |
| Creatinine, mg/dL | 0.457 (0.122–1.707) |  |  | |
| Total cholesterol, mg/dL | 1.005 (0.999–1.011)^†^ | 1.001 (0.993–1.010) | 1.004 (0.997–1.011) | |
| Triglyceride, mg/dL | 1.002 (0.999–1.005) |  |  | |
| High-density lipoprotein, mg/dL | 1.006 (0.979–1.035) |  |  | |
| Low-density lipoprotein, mg/dL | 1.006 (0.998–1.013) |  |  | |
| HbA1c, % | 1.174 (0.967–1.424) |  |  | |
| Fasting glucose, mg/dL | 1.003 (1.003–1.019)^*^ | 0.995 (0.983–1.007) | 0.995 (0.983–1.007) | |
| Fibrinogen, mg/dL | 0.998 (0.994–1.002) |  |  | |
| Blood viscosity, centipoise |  |  |  | |
| Low shear viscosity | 1.241 (1.073–1.435)^*^ | 1.285 (1.010–1.634)^*^ | 1.326 (1.049–1.676)^*^ | |
| High shear viscosity | 1.567 (0.909–2.702) |  |  | |

Data are shown as odds ratio (95% confidence interval).

CI, confidence interval; NIHSS, National Institutes of Health Stroke Scale; OR, odds ratio.

^*^p < 0.05, ^†^p < 0.1

^a^Adjusted for history of hypertension, history of diabetes mellitus, prior antithrombotics use, initial NIHSS score, systolic blood pressure, diastolic blood pressure, hemoglobin, blood urea nitrogen, total cholesterol, fasting glucose, and low shear viscosity.

^b^Adjusted for history of hypertension, history of diabetes mellitus, history of stroke, initial NIHSS score, systolic blood pressure, diastolic blood pressure, hemoglobin, blood urea nitrogen, total cholesterol, fasting glucose, and low shear viscosity.

**Supplementary Table S11.** Comparison of clinical and laboratory findings according to early neurological deterioration after index stroke caused by local branch occlusion

|  | END (-) (n = 48) | END (+) (n = 20) | *P* |
| --- | --- | --- | --- |
| Demographics |  |  |  |
| Sex, male | 14 (29.2) | 16 (80.0) | <0.001 |
| Age, years | 73.0 ± 10.8 | 71.8 ± 9.8 | 0.669 |
| Risk factors |  |  |  |
| Hypertension | 42 (87.5) | 16 (80.0) | 0.465 |
| Diabetes mellitus | 20 (41.7) | 6 (30.0) | 0.367 |
| Hyperlipidemia | 36 (75.0) | 10 (50.0) | 0.045 |
| Smoking | 10 (20.8) | 6 (30.0) | 0.417 |
| History of stroke | 9 (18.8) | 8 (40.0) | 0.065 |
| Prior medication |  |  |  |
| Antithrombotics | 28 (58.3) | 4 (20.0) | 0.007 |
| Statins | 24 (50.0) | 2 (10.0) | 0.002 |
| Initial NIHSS score | 3.5 [2.0 – 6.8] | 4 [3.0 – 4.0] | 0.892 |
| Systolic blood pressure, mmHg | 153.3 ± 24.9 | 153.3 ± 22.6 | 0.999 |
| Diastolic blood pressure, mmHg | 83.7 ± 12.7 | 81.2 ± 7.3 | 0.312 |
| Blood laboratory findings |  |  |  |
| White blood cell count, x10^9^/L | 7.7 ± 2.2 | 6.6 ± 1.6 | 0.062 |
| Hematocrit, % | 39.0 ± 7.2 | 42.3 ± 4.3 | 0.061 |
| Hemoglobin, mg/dL | 13.1 ± 2.5 | 14.2 ± 1.6 | 0.070 |
| Platelet count, x10^9^/L | 233.6 ± 75.0 | 214.6 ± 37.5 | 0.171 |
| Blood urea nitrogen, mg/dL | 16.9 ± 7.6 | 21.4 ± 8.6 | 0.037 |
| Creatinine, mg/dL | 0.8 ± 0.3 | 0.8 ± 0.2 | 0.738 |
| Total cholesterol, mg/dL | 180.7 ± 61.7 | 194.6 ± 39.0 | 0.270 |
| Triglyceride, mg/dL | 133.1 ± 61.5 | 147.6 ± 71.7 | 0.403 |
| High-density lipoprotein, mg/dL | 51.8 ± 14.3 | 50.3 ± 8.0 | 0.576 |
| Low-density lipoprotein, mg/dL | 105.3 ± 51.4 | 118.5 ± 27.0 | 0.171 |
| HbA1c, % | 6.6 ± 1.4 | 6.6 ± 1.7 | 0.920 |
| Fasting glucose, mg/dL | 131.1 ± 53.4 | 120.4 ± 35.7 | 0.415 |
| Fibrinogen, mg/dL | 316.3 ± 91.0 | 305.6 ± 113.6 | 0.682 |
| Blood viscosity, centipoise |  |  |  |
| Low shear viscosity | 11.6 [10.3 – 13.0] | 13.0 [11.4 – 17.1] | 0.011 |
| High shear viscosity | 3.8 [3.5 – 4.0] | 4.0 [3.7 – 5.1] | 0.016 |

Data are presented as n (%), mean ± standard deviation, or median [interquartile range].

END, early neurological deterioration; NIHSS, National Institutes of Health Stroke Scale

**Supplementary Table S12.** Associated factors for early neurological deterioration after ischemic stroke caused by local branch occlusion.

| Characteristic | Crude OR (95% CI) | Adjusted OR (95% CI)^a^ | Adjusted OR (95% CI)^b^ |
| --- | --- | --- | --- |
| Demographics |  |  |  |
| Sex, male | 9.714 (2.755–34.250)^*^ | 3.224 (0.642–16.177) | 2.161 (0.395–11.826) |
| Age, years | 0.989 (0.941–1.040) |  |  |
| Risk factors |  |  |  |
| Hypertension | 0.571 (0.142–2.294) |  |  |
| Diabetes mellitus | 0.600 (0.197–1.830) |  |  |
| Hyperlipidemia | 0.333 (0.112–0.995)^*^ | 0.203 (0.029–1.412) | 0.262 (0.048–1.419) |
| Smoking | 1.629 (0.499–5.317) |  |  |
| History of stroke | 2.889 (0.913–9.136)^†^ |  | 4.822 (0.372–62.508) |
| Prior medication |  |  |  |
| Antithrombotics | 0.179 (0.052–0.615)^*^ | 3.542 (0.230–54.504) |  |
| Statins | 0.111 (0.023–0.532)^*^ | 0.106 (0.004–2.587) | 0.129 (0.008–2.054) |
| Initial NIHSS score | 0.979 (0.807–1.187) |  |  |
| Systolic blood pressure, mmHg | 1.000 (0.978–1.022) |  |  |
| Diastolic blood pressure, mmHg | 0.981 (0.937–1.027) |  |  |
| Blood laboratory findings |  |  |  |
| White blood cell count, x10^9^/L | 0.760 (0.565–1.021)^†^ | 0.808 (0.469–1.315) | 0.855 (0.540–1.356) |
| Hematocrit, % | 1.115 (0.991–1.253)^†^ |  |  |
| Hemoglobin, mg/dL | 1.334 (0.967–1.841)^†^ | 1.326 (0.962–1.828)^†^ | 1.231 (0.898–1.689) |
| Platelet count, x10^9^/L | 0.996 (0.988–1.004) |  |  |
| Blood urea nitrogen, mg/dL | 1.069 (1.001–1.142)^*^ | 1.018 (0.928–1.117) | 1.034 (0.942–1.135) |
| Creatinine, mg/dL | 0.693 (0.084–5.698) |  |  |
| Total cholesterol, mg/dL | 1.004 (0.995–1.014) |  |  |
| Triglyceride, mg/dL | 1.003 (0.995–1.012) |  |  |
| High-density lipoprotein, mg/dL | 0.990 (0.949–1.033) |  |  |
| Low-density lipoprotein, mg/dL | 1.006 (0.995–1.018) |  |  |
| HbA1c, % | 1.019 (0.716–1.450) |  |  |
| Fasting glucose, mg/dL | 0.995 (0.983–1.007) |  |  |
| Fibrinogen, mg/dL | 0.999 (0.993–1.004) |  |  |
| Blood viscosity, centipoise |  |  |  |
| Low shear viscosity | 1.563 (1.173–2.083)^*^ | 1.524 (1.035–2.246)^*^ | 1.362 (0.929–1.997) |
| High shear viscosity | 5.725 (1.796–18.255)^*^ |  |  |

Data are shown as odds ratio (95% confidence interval).

CI, confidence interval; NIHSS, National Institutes of Health Stroke Scale; OR, odds ratio.

^*^p < 0.05, ^†^p < 0.1

^a^Adjusted for sex, history of hyperlipidemia, antithrombotics use, prior statins use, prior white blood cell count, hemoglobin, blood urea nitrogen, and low shear viscosity.

^b^Adjusted for sex, history of hyperlipidemia, history of stroke, prior statins use, white blood cell count, hemoglobin, blood urea nitrogen, and low shear viscosity.
